# Effects of Soil Pre-Treatment with Basamid® Granules, *Brassica juncea, Raphanus sativus*, and *Tagetes patula* on Bacterial and Fungal Communities at Two Apple Replant Disease Sites

**DOI:** 10.3389/fmicb.2017.01604

**Published:** 2017-09-01

**Authors:** Bunlong Yim, Heike Nitt, Andreas Wrede, Samuel Jacquiod, Søren J. Sørensen, Traud Winkelmann, Kornelia Smalla

**Affiliations:** ^1^Institute of Horticultural Production Systems, Leibniz Universität Hannover Hannover, Germany; ^2^Federal Research Centre for Cultivated Plants (JKI), Institute for Epidemiology and Pathogen Diagnostics Braunschweig, Germany; ^3^Department of Plant Production, Plant Protection, Environment, Landwirtschaftskammer Schleswig-Holstein Ellerhoop, Germany; ^4^Department of Horticulture, Landwirtschaftskammer Schleswig-Holstein Ellerhoop, Germany; ^5^Section of Microbiology, Department of Biology, University of Copenhagen Copenhagen, Denmark

**Keywords:** amplicon sequencing, apple replant disease, biofumigation, soil microbiome

## Abstract

Nurseries producing apple and rose rootstock plants, apple orchards as well as rose production often experience replanting problems after several cultivations at the same site when a chemical soil disinfectant is not applied. The etiology of apple and rose replanting problems is most likely caused by soil-borne pathogen complex, defined as “replant disease (RD)”. Symptoms typical of RD are reduced shoot and root growth, a smaller leaf area, a significant decrease in plant biomass, yield and fruit quality and a shorter life span. In our previous study, we showed that RD symptoms were reduced when apple rootstock M106 were grown in RD soils treated either with the soil fumigant Basamid or after biofumigation by incorporating *Brassica juncea* or *Raphanus sativus* or by growing *Tagetes* under field conditions compared to untreated control soil. The present study aimed at identifying potential bacterial and fungal taxa that were affected by different soil treatments and linking bacterial and fungal responders to plant performance. Miseq® Illumina® sequencing of 16S rRNA gene fragments (bacteria) and ITS regions (fungi) amplified from total community DNA extracted from soil samples taken 4 weeks after treatments were performed. Soil properties and culture history of the two RD sites greatly influenced soil microbiomes. Several bacterial genera were identified that significantly increased in treated soils such as *Arthrobacter* (*R. sativus*, both sites), *Curtobacterium* (Basamid, both sites), *Terrimonas* (Basamid and *R. sativus*, site A) and *Ferruginibacter* (*B. juncea*, site K and *R. sativus*, site A) that were also significantly and positively correlated with growth of apple M106 plants. Only few fungal genera, such as *Podospora, Monographella* and *Mucor*, were significantly promoted in soils treated with *B. juncea* and *R. sativus* (both sites). The least pronounced changes were recorded for bacterial as well as fungal communities in the RD soils planted with *Tagetes*. The detection of bacterial and fungal genera that were significantly increased in relative abundance in response to the treatments and that were positively correlated with plant growth suggests that management of the soil microbial community could contribute to overcome the apple RD encountered at affected sites.

## Introduction

The soil microbiome is assumed to play a crucial role for plant growth and health in terms of acquiring water and nutrients, acting antagonistically against soil-borne plant pests and pathogens, as well as inducing plant defense responses against pathogens (Berendsen et al., 2012). Negative effects of the soil microbiome on plant growth and yield were also revealed, especially at sites with monocultures and with lack of sustainable management practices (Magarey, 1999; Seigies and Pritts, 2006; Wu et al., 2015; Zhao et al., 2016). This is likely due to a reduced microbial diversity because of the repeated monoculturing (Howe et al., 2014).

Apple plants cultivated repeatedly at the same site have often been reported to show reduced shoot and root growth. It is assumed that pathogenic microorganisms increased in abundance in response to plant root exudations of previous cultures (Badri and Vivanco, 2009; Mazzola and Manici, 2012; Yim et al., 2013; Nicola et al., 2017). This so-called apple replant disease (ARD) has severe consequences in terms of economic losses in tree nurseries and apple production worldwide.

A recent study employing transcriptomic analysis in roots of apple rootstock M26 plants grown in ARD soils compared to Gamma-sterilized soil discovered that the expression of plant genes associated with plant defense, i.e., phytoalexin production genes was increased while genes involved in the primary metabolism were less expressed (Weiß et al., 2017) indicating plant response to soil-borne pathogens. Possible ARD causing organisms identified from cultivation dependent approaches included actinomycetes (Otto et al., 1994), *Pythium* sp. (Hoestra, 1994; Emmett et al., 2014), *Cylindrocarpon* sp., *Phytophthora* sp., *Rhizoctonia solani* (Mazzola, 1998; Tewoldemedhin et al., 2011; Kelderer et al., 2012) and nematodes, e.g., the soil endoparasitic nematode *Pratylenchus penetrans* (Mai et al., 1994). Several recent studies employed total community (TC-) DNA-based approaches to identify these pathogens, but rather showed microbial community shifts in ARD soils after soil treatments that restored apple growth (Yim et al., 2013; Sun et al., 2014; Franke-Whittle et al., 2015; Nicola et al., 2017). Because the etiology of ARD is complex, conventional soil fumigants with a broad spectrum of biocides such as chloropicrin, 1.2 dichloropropane, 1.3 dichloropropene, methyl bromide and Basamid® granules were shown to be the most effective treatments against ARD (Mai and Abawi, 1978; Brown and Koutoulis, 2008; Yim et al., 2013; Nicola et al., 2017). However, those chemical substances were reported to be toxic, and their application is no longer allowed in many countries (Ruzo, 2006; Porter et al., 2010).

For environmentally friendly approaches, crop rotation or treating replant disease (RD) soil using several Brassicaceae species (biofumigation) or *Tagetes* (nematode repelling) demonstrated promising effects against disease-causing organisms in soils (Sarwar et al., 1998; Topp et al., 1998; Mattner et al., 2008; Marahatta et al., 2012; Pino et al., 2016), and subsequently reduced RD symptoms on plant growth (Seigies and Pritts, 2006; Mazzola et al., 2015; Yim et al., 2016). Effects of biofumigation originate from plant secondary metabolites glucosinolates (GS) that are hydrolyzed mainly by plant myrosinase enzymes (reviewed by Halkier and Gershenzon, 2006), subsequently releasing several compounds depending on soil properties (Halkier and Gershenzon, 2006), such as isothiocyanates (ITC), nitriles, thiocyanates, epithionitriles, and oxazolidine-2-thiones (Brown et al., 1991; Kirkegaard and Sarwar, 1998). Among GS-degraded products, volatile ITCs were shown to be responsible for suppression of weeds (Sarwar et al., 1998; Malik et al., 2008; Mattner et al., 2008), soil-borne plant pests and pathogens in different crop systems (Borek et al., 1998; Peterson et al., 1998; Matthiessen and Shackleton, 2005; Bones and Rossiter, 2006; Mazzola et al., 2007; Mattner et al., 2008; Aires et al., 2009; Agerbirk and Olsen, 2012; Neubauer et al., 2014). On the other hand, *Tagetes* plants are renowned to exhibit toxicity in soils due to their thiophene contents (Hooks et al., 2010; Saha et al., 2012). Highly suppressed growth of several soil-borne plant pathogenic fungi such as *R. solani* and *Fusarium solani* mediated by these biocidal compounds was demonstrated via *in vitro* evaluations (Saha et al., 2012).

In our previous field study, the effects of pre-treatments of RD soils with the soil fumigant Basamid, biofumigation with *Brassica juncea* and *Raphanus sativus* and growing *Tagetes* plants at the two sites K and A on plant performance were investigated. Findings revealed that effects of the different treatments evaluated by field growth of apple rootstock M106 plants were site-dependent. At site K, shoot fresh mass (SFM) of the M106 plants significantly increased by 155, 148, 165, and 175% in treated soils with Basamid, *B. juncea, R. sativus*, and *Tagetes*, respectively, relative to the corresponding RD soil. At site A, a moderate effect was observed only for the RD soil cropped with *Tagetes*, with 52% increment in SFM (Yim et al., 2016). Changes in the bacterial and fungal community composition based on DGGE fingerprint analysis revealed a treatment- and site-dependent pattern (Yim et al., 2016), calling for deeper molecular investigations and characterization of these differences.

In the present study, a detailed analysis of the changes of soil bacterial and fungal community composition at the two sites was performed, focusing on diversity and relative abundances at different taxonomic levels in response to the treatments by means of Miseq® Illumina® sequencing. This study identified soil bacterial and fungal taxa affected by the different soil treatments (Basamid, *B. juncea, R. sativus*, and *Tagetes*) at the two sites under field conditions, and linked these microbial responders to ARD suppression.

## Materials and methods

The two RD sites K (53° 41′ 58.51″ N, 9° 41′ 34.12″ E) and A (53° 42′ 18.81″ N, 9° 48′ 16.74″ E) that had been used for producing rose and apple rootstocks, respectively, were submitted to different treatments under field conditions during the years 2012 and 2013 with permission by the owners. The sites differ in soil chemical and physical properties as described in Yim et al. (2016). Briefly, site K (sandy soil) has a higher proportion in organic matter and sand than site A (slightly loamy sand). Five treatments and three biological replicates (plots) per treatment were randomized in blocks on an area of 1,000 m^2^ per site (45 m^2^ per replicate). Parcels replanted with apple rootstocks M4 and M111 in May 2012 and 2013, respectively, served as untreated RD soils. The rootstocks were harvested each year in November. For treatment with Brassicaceae plants, seeds from two species, *B. juncea* ‘Terra Plus’ (12 kg ha^−1^) and *R. sativus* ‘Defender’ (30 kg ha^−1^) were sown onto RD soils twice, in April/May and in June/July (2012 and 2013). The plants at full flowering, about 8 weeks after sowing were cut at the soil line, chopped and subsequently incorporated into the soils using Humus WM Flail mulchers (Humus®, Bermatingen, Germany) and a common rotary cultivator (Yim et al., 2016). For treatment with *Tagetes patula* ‘Nemamix,’ 10 kg ha^−1^ seeds were sown once per year in 2012 and 2013, in April/May. In both years, the plants grew until November before they were plowed. Seeds of *B. juncea, R. sativus*, and *Tagetes* were supplied by P. H. Petersen Saatzucht Lundsgaard GmbH, Germany. A chemical soil fumigant treatment with Basamid® granules (97% Dazomet) was performed once in August 2013 at a dose of 400 kg ha^−1^ (ProfiFlor GmbH, Stommeln, Germany) applied when the second biofumigation was carried out (end of August 2013).

Four weeks after the Basamid and biofumigation treatments, bulk soils were sampled the same day in September 2013 using a 3.5 cm diameter core soil sampler at 0–20 cm depth. The sampling schedule and procedures were the same as for the treatments with *Tagetes* and untreated RD. At the sampling date, the flowering *Tagetes* plants had not been incorporated into the soil. The homogenized and sieved (mesh sizes ≤ 2 mm) soil samples were submitted to TC-DNA extraction and purification as described in Yim et al. (2016). In brief, 0.5 g of soil was used for TC-DNA extraction after a harsh cell lysis.

Amplicon sequencing for bacteria and fungi was implemented via Miseq® Illumina® (Illumina, San Diego, CA, USA) sequencing. For the bacterial 16S rRNA gene fragments, an initial PCR amplification step was performed using a set of primer pairs 341F (CCTAYGGGRBGCASCAG) and 806R (GGACTACHVGGGTWTCTAAT) to flank the approximate 460 bp variable V3-V4 regions as described by Nunes et al. (2016). Regarding the ITS regions for fungi, primers gITS7 (GTGARTCATCGARTCTTTG) and ITS4 (TCCTCCGCTTATTGATATGC) were applied to obtain the fragments of interest (Ihrmark et al., 2012). Purification and size-selection of products of more than 100 bp from a second amplification step using the same primers with attachment of adaptors and barcode tags was performed with Agencourt AMPure XP beads (Beckman Coulter, Brea, CA, USA) according to the manufacturer's instructions. The samples were then pooled and adjusted to equimolar concentrations measured using a Qubit Fluorometer (Life Technologies, Carlsbad, CA, USA), concentrated using the DNA Clean and Concentrator™-5 kit (Zymo Research, Irvine, CA, USA), and finally subjected to 2 × 250 bp paired-end high-throughput sequencing on an Illumina® MiSeq® platform.

Amplicon sequences were analyzed using qiime_pipe (https://github.com/maasha/qiime_pipe) with default settings, which performs sample demultiplexing, quality-based sequence trimming, primer removal and paired-end reads assembly prior to annotation workflow (Caporaso et al., 2010). Annotation procedure for bacterial sequences is derived from previously described work (Nunes et al., 2016). Chimera check was done with UCHIME (Edgar et al., 2011) and Operational Taxonomic Units (OTUs) were picked at 97% sequence identity level. OTU representative sequences were selected by the highest abundance within the cluster and assigned to taxonomy using the RDP classifier, with a confidence threshold of 80%. Read contingency tables were exported at the species level in order to define OTUs. For fungi, if a sequence had the same bit score to more than one species hypothesis (SH) in the UNITE version 7.0 database (Koljalg et al., 2013) of Megablast (Camacho et al., 2009), then it was assigned to the most abundant SH in the dataset. Selected OTUs were based on the assigned sequences that were more than 95% similarity to any SH or had greater than 100 bp alignment length. Illumina sequencing data were deposited at the NCBI sequence read archive under the accession number PRJNA352771.

### Data analyses

For subsequent analyses, three biological replicates were used for bacteria, and four replicates for fungi, except for the treatment with *Tagetes* for which only three replicates could be employed. The excluded replicates of the respective treatments were based on high variability of the sequence reads (two to three time differences). The effects of the different soil treatments on bacterial and fungal community compositions were analyzed by a Principal Coordinate Analysis (PCoA) applying Bray-Curtis distance metrics and the analysis of similarity (ANOSIM) test by Past3 (3.02) (Hammer et al., 2001). Species richness and diversity index were evaluated using rarefied sequence data applying Tukey test adapted based on Herberich et al. (2010) at *p* < 0.05 with transformed data by sqrt(n/N ^*^ 100 +1) (n, the number of sequences for each OTU and N, the total number of sequences from the sample) to reveal significant differences in relative abundances of soil bacteria and fungi at phylum levels (software R 3.2.2). Any bacterial and fungal genera that presented significant differences in their relative abundances between the soil treatments, and those which were greater than 0.5% relative abundance were tested for correlation with shoot and root fresh mass of apple rootstock M106 plants grown in the field in 2014, using the Pearson correlation coefficient (r) by Past3 (3.02).

## Results

### Effects of treatments on soil bacterial community composition and diversity

**Figure 1:**
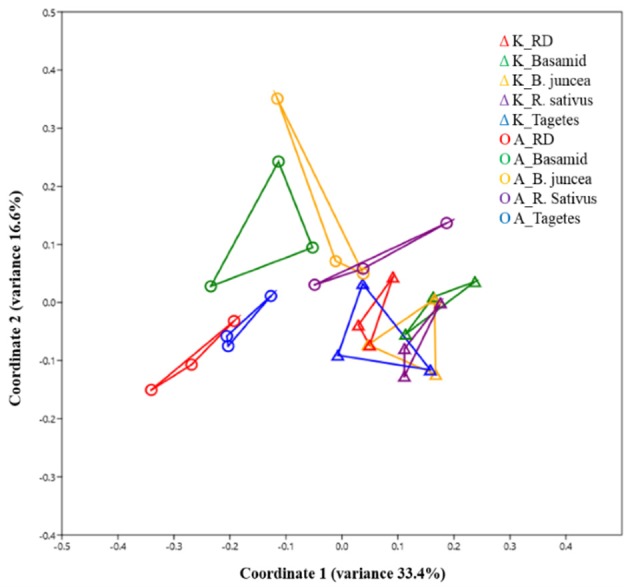
Effect of different treatments on soil bacterial community composition under field conditions revealed by principal coordinate analysis (PCoA) using Bray-Curtis distance metric. Past3 and *n* = 3. Soil samples were taken 4 weeks after different treatments in September 2013.

The numbers of bacterial sequences detected ranged from 18,576 to 27,738 and from 21,267 to 40,089 in soils at sites K and A, respectively, with no significant differences between the treatments. However, a tendency for higher sequence counts was observed in untreated RD soils rather than in the other treatments at both sites (Table 1). Subsequent analyses using rarefied sequence data recorded more OTUs in soils treated with *B. juncea* (sites K, 347 and A, 302) and *R. sativus* (sites K, 353 and A, 340) than in soils subjected to the other treatments. Except that significantly higher species richness in *R. sativus*-treated soil at site A was observed, bacterial compositions and diversities were not significantly altered by the treatments in soils at both sites (numbers of OTUs, Chao1 and Shannon indices, Table 1) in comparison to untreated RD soils. The bacterial diversities were significantly lower in soils at site A than K, regardless of different soil treatments (Table S2; Figure S1). Analyses of similarity (ANOSIM) indicated significantly distinct bacterial community compositions between sites (*R* = 0.46, *p* < 1E-4, Table 2), irrespective of the treatment. Both PCoA and ANOSIM tests revealed that the bacterial community composition in soil of the *Tagetes* treatment at site A was less affected compared to the other treatments (Figure 1; Table 2). Overall, the soil treatments resulted in stronger alterations of the bacterial community composition at site A than at site K (*R*-values, Table 2; PCoA, Figure 1). In addition, for soil samples from the *R. sativus* treatments at site A, the highest *R*-value (0.74) was recorded (Table 2).

**Table 1:** Bacterial community diversity based on operational taxonomic units (OTUs) at 97% similarity in different soil treatments.

**Site**	**Treatment**	**Sequences per condition**	**Numbers of OTU (97%)**	**Chao1**	**Shannon**
K	K_RD	27,738 ± 2,755	332 ± 16 ab	368 ± 18 ab	4.18 ± 0.12
	K_Basamid	18,576 ± 3,728	311 ± 5 a	350 ± 7 a	4.30 ± 0.02
	K_*B. juncea*	24,632 ± 3,770	347 ± 3 ab	395 ± 14 b	4.36 ± 0.02
	K_*R. sativus*	26,946 ± 4,508	353 ± 1 b	389 ± 6 ab	4.29 ± 0.05
	K_*Tagetes*	25,259 ± 3,909	327 ± 7 ab	362 ± 7 ab	4.13 ± 0.10
A	A_RD	40,089 ± 7,422	284 ± 13 a	317 ± 18 a	3.69 ± 0.11
	A_Basamid	32,016 ± 2,551	274 ± 20 a	308 ± 18 a	3.74 ± 0.17
	A_*B. juncea*	30,793 ± 8,640	302 ± 31 ab	360 ± 15 ab	3.51 ± 0.65
	A_*R. sativus*	21,267 ± 3,228	340 ± 6 b	383 ± 14 b	4.14 ± 0.05
	K_*Tagetes*	29,665 ± 2,160	293 ± 3 a	349 ± 16 ab	3.84 ± 0.04

*Data is presented as mean ± SEM. RD, replant disease soil. Letters indicate significant differences within site, Tukey test p < 0.05 and n = 3. Chao1, species richness. Within site, increased bacterial richness and diversity in treated RD soils compared to untreated are highlighted in green*.

**Table 2:** Analysis of similarities of the bacterial community composition detected in different soil treatments with respect to untreated replant disease soil based on OTUs of bacterial 16S rRNA gene fragments.

**Treatment**	**Site K**		**Site A**	
	***R*-value**	***p*-value**	***R*-value**	***p*-value**
Basamid	0.48	0.2015	0.56	0.0948
*B. juncea*	0.22	0.4032	0.48	0.1016
*R. sativus*	0.30	0.2949	0.74	0.1003
*Tagetes*	−0.26	0.9056	0.07	0.5998

*For sites K vs. A, R-value = 0.46 and p < 0.0001. R- (−1 to 1) and p-values were obtained from ANOSIM-test. R-value close to “1” suggests strong dissimilarity between the communities being compared, whereas the value close to “0” represents an even distribution of the communities within and between treatments. The R-value below “0” suggests that dissimilarities are greater within treatment than between treatments*.

**Figure 2:**
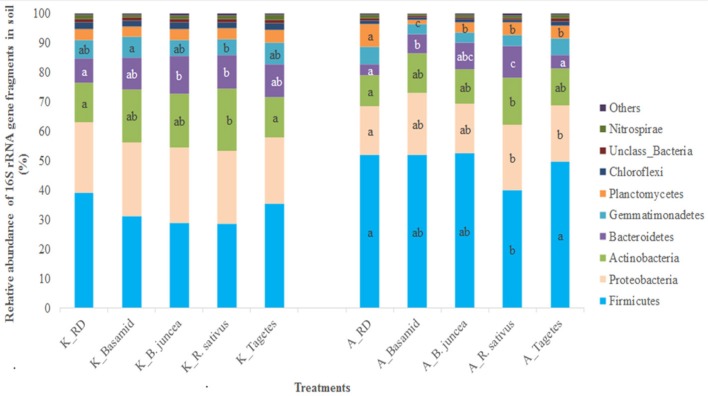
Relative abundance of dominant bacterial phyla in soils at the two sites affected by the different treatments. Different letters within the phylum indicate significant differences between soil treatments within site, Tukey test, *p* < 0.05 and *n* = 3.

Among the analyzed samples, 12 bacterial phyla were identified, and *Firmicutes* were most dominant in relative abundance, followed by *Proteobacteria* and *Actinobacteria* in all soil treatments and at both sites (Figure 2; Table S3). *Firmicutes* shared proportions of about 29–39% in soils at site K, but higher abundances of approximately 40–52% at site A (Figure 2). Members of the bacterial phyla *Actinobacteria* and *Bacteroidetes* were observed in significantly higher relative abundances in soils treated with *R. sativus* compared with untreated RD soils at both sites, K and A. Site-dependent effects of the treatments on other bacterial phyla were detected. For instance, the relative abundance of *Proteobacteria* was significantly higher in *R. sativus* and *Tagetes* than in untreated RD soils only at site A (Figure 2). Another bacterial phylum, *Planctomycetes*, was significantly reduced only in soils at site A when the RD soil was treated with Basamid, *B. juncea, R. sativus*, and *Tagetes*. At site K, treatments with Basamid and *Tagetes* did not significantly affect members of any bacterial phylum (Figure 2).

At genus level, soils fumigated with Basamid exhibited the following increased common responders in relative abundance: *Salinibacterium, Curtobacterium, Thiobacillus*, and *Rhodanobacter* with the strongest response (33- and 23-fold increase at sites K and A, respectively) recorded for *Rhodanobacter*. Only the unclassified *Bacteroidales*-related sequences significantly decreased in relative abundance in Basamid-treated soils at both sites (Table 3).

**Table 3:** Relative abundance of bacterial genera detected in TC-DNAs extracted from bulk soils taken 4 weeks after different treatments at two replant disease sites (only genera with a relative abundance > 0.5 % are shown).

**Phylum/Family**	**Genus**	**Site K**					**Site A**				
		**K_RD**	**K_Basamid**	**K_*B. juncea***	**K_*R. sativus***	**K_*Tagetes***	**A_RD**	**A_Basamid**	**A_*B. juncea***	**A_*R. sativus***	**A_*Tagetes***
***Actinobacteria***											
*Micrococcaceae*		2.04 ± 0.53 a	4.02 ± 0.79 ab	6.14 ± 0.43 b	8.95 ± 0.43 c	1.80 ± 0.18 a	0.95 ± 0.09 a	4.82 ± 0.24 b	2.38 ± 0.61 ab	4.49 ± 1.14 bc	1.32 ± 0.06 ac
	*Arthrobacter*	1.92 ± 0.51 a	3.64 ± 0.64 ab	5.89 ± 0.34 b	8.61 ± 0.41 c	1.70 ± 0.18 a	0.92 ± 0.09 a	2.50 ± 0.80 ab	2.30 ± 0.61 ab	4.33 ± 1.06 b	1.31 ± 0.07 a
*Microbacteriaceae*		0.16 ± 0.02 a	1.12 ± 0.23 b	0.24 ± 0.03 a	0.30 ± 0.01 a	0.25 ± 0.05 a	0.07 ± 0.02 a	0.79 ± 0.25 b	0.11 ± 0.03 a	0.16 ± 0.01 a	0.09 ± 0.01 a
	*Salinibacterium*	0.07 ± 0.02 a	0.59 ± 0.14 b	0.11 ± 0.01 a	0.12 ± 0.01 a	0.13 ± 0.02 a	0.04 ± 0.01 a	0.62 ± 0.23 b	0.05 ± 0.02 a	0.06 ± 0.02 a	0.05 ± 0.01 a
	*Curtobacterium*	0.08 ± 0.01 a	0.54 ± 0.09 b	0.13 ± 0.02 a	0.17 ± 0.01 a	0.11 ± 0.02 a	0.03 ± 0.01 a	0.17 ± 0.03 b	0.06 ± 0.02 ab	0.09 ± 0.01 ab	0.04 ± 0.01 a
*Intrasporangiaceae*		0.53 ± 0.04 a	0.59 ± 0.10 a	1.23 ± 0.21 b	1.33 ± 0.17 b	0.48 ± 0.04 a	0.51 ± 0.09 a	0.45 ± 0.16 a	0.83 ± 0.18 ab	1.29 ± 0.10 b	0.69 ± 0.12 ab
	*Terrabacter*	0.27 ± 0.02 a	0.29 ± 0.04 a	0.73 ± 0.14 b	0.86 ± 0.11 b	0.22 ± 0.01 a	0.36 ± 0.08 a	0.26 ± 0.10 a	0.58 ± 0.13 ab	0.92 ± 0.07 b	0.49 ± 0.07 a
*Streptomycetaceae*		1.01 ± 0.13 a	0.67 ± 0.09 ab	0.46 ± 0.01 b	0.47 ± 0.02 b	0.39 ± 0.02 b	0.57 ± 0.10 ab	0.39 ± 0.02 a	0.56 ± 0.16 ab	0.69 ± 0.01 b	0.52 ± 0.05 ab
	*Streptomyces*	0.60 ± 0.14 a	0.14 ± 0.02 b	0.14 ± 0.03 b	0.13 ± 0.02 b	0.12 ± 0.00 b	0.05 ± 0.02	0.04 ± 0.00	0.07 ± 0.02	0.08 ± 0.01	0.03 ± 0.00
***Bacteroidetes***											
*Chitinophagaceae*		6.04 ± 0.20 a	8.22 ± 0.86 ab	9.26 ± 0.49 b	7.87 ± 0.17 b	8.05 ± 1.07 ab	2.09 ± 0.14 a	5.38 ± 0.23 b	7.68 ± 2.33 abc	9.20 ± 0.20 c	2.92 ± 0.29 a
	*Terrimonas*	2.79 ± 0.11	3.62 ± 0.19	3.65 ± 0.27	3.56 ± 0.22	3.74 ± 0.43	0.47 ± 0.11 a	1.50 ± 0.20 b	1.85 ± 0.71 abc	2.49 ± 0.13 c	0.95 ± 0.09 ab
	*Ferruginibacter*	1.03 ± 0.05 a	1.31 ± 0.27 ab	1.91 ± 0.04 b	1.38 ± 0.09 a	1.23 ± 0.09 a	0.25 ± 0.03 a	1.14 ± 0.36 ab	1.05 ± 0.34 ab	1.33 ± 0.11 b	0.43 ± 0.09 a
	*Flavitalea*	0.24 ± 0.03	0.28 ± 0.04	0.33 ± 0.02	0.27 ± 0.05	0.41 ± 0.13	0.34 ± 0.02 a	0.55 ± 0.14 ab	1.21 ± 0.41 ab	1.20 ± 0.30 b	0.54 ± 0.04 b
*Unclass_Bacteroidales*	*Unclass_Bacteroidales*	0.85 ± 0.26 a	0.27 ± 0.01 b	1.40 ± 0.47 a	0.99 ± 0.08 a	0.89 ± 0.14 a	0.88 ± 0.00 a	0.14 ± 0.03 b	0.48 ± 0.10 ab	0.53 ± 0.12 a	0.73 ± 0.07 a
*Flavobacteriaceae*	*Unclass_Flavobacteriaceae*	0.34 ± 0.05 a	1.35 ± 0.16 b	0.61 ± 0.18 ab	0.46 ± 0.01 a	0.54 ± 0.07 a	0.29 ± 0.04	0.34 ± 0.05	0.31 ± 0.09	0.42 ± 0.10	0.22 ± 0.01
***Planctomycetes***											
*Planctomycetaceae*	*Unclass_Planctomycetaceae*	3.70 ± 1.35	3.51 ± 0.19	3.67 ± 0.81	3.53 ± 0.28	4.28 ± 1.11	7.60 ± 0.57 a	1.50 ± 0.08 b	3.65 ± 0.84 c	4.06 ± 0.22 c	4.34 ± 0.75 c
***Alphaproteobacteria***											
*Rhizobiaceae*		0.47 ± 0.08 ab	0.17 ± 0.03 a	0.63 ± 0.02 b	0.77 ± 0.12 b	0.36 ± 0.07 ab	0.12 ± 0.03 a	0.07 ± 0.04 a	0.33 ± 0.17 ab	0.49 ± 0.09 b	0.16 ± 0.01 a
	*Rhizobium*	0.38 ± 0.12 ab	0.08 ± 0.01 a	0.52 ± 0.02 b	0.61 ± 0.09 b	0.30 ± 0.06 b	0.08 ± 0.02 a	0.05 ± 0.02 a	0.26 ± 0.13 ab	0.36 ± 0.07 b	0.14 ± 0.01 ab
*Sphingomonadaceae*		2.52 ± 0.24 ab	3.40 ± 0.27 b	2.73 ± 0.14 ab	2.43 ± 0.12 ab	1.92 ± 0.18 a	1.14 ± 0.09	1.39 ± 0.20	1.43 ± 0.33	1.79 ± 0.20	1.60 ± 0.31
	*Sphingomonas*	0.05 ± 0.02 a	0.51 ± 0.12 b	0.03 ± 0.01 a	0.03 ± 0.00 a	0.03 ± 0.01 a	0.03 ± 0.01	0.22 ± 0.13	0.01 ± 0.00	0.03 ± 0.01	0.00 ± 0.00
***Betaproteobacteria***											
*Oxalobacteraceae*	*Massilia*	0.24 ± 0.06 a	0.88 ± 0.04 b	0.23 ± 0.02 a	0.25 ± 0.02 a	0.15 ± 0.03 a	0.07 ± 0.01	0.16 ± 0.07	0.16 ± 0.04	0.22 ± 0.05	0.27 ± 0.07
*Hydrogenophilaceae*	*Thiobacillus*	0.21 ± 0.01 a	0.54 ± 0.12 b	0.21 ± 0.03 ab	0.24 ± 0.02 ab	0.22 ± 0.02 ab	0.11 ± 0.02 a	0.86 ± 0.12 b	0.25 ± 0.09 ac	0.21 ± 0.04 ac	0.25 ± 0.01 c
***Gammaproteobacteria***											
*Xanthomonadaceae*		0.91 ± 0.08 a	2.29 ± 0.05 b	1.62 ± 0.14 c	1.60 ± 0.17 abc	1.09 ± 0.13 ac	1.01 ± 0.23	4.18 ± 1.63	1.11 ± 0.23	1.82 ± 0.27	0.97 ± 0.05
	*Rhodanobacter*	0.05 ± 0.01 a	1.65 ± 0.12 b	0.22 ± 0.12 a	0.12 ± 0.03 a	0.05 ± 0.02 a	0.15 ± 0.06 a	3.49 ± 1.57 b	0.10 ± 0.03 a	0.22 ± 0.10 a	0.07 ± 0.02 a
*Pseudomonadaceae*		1.93 ± 0.25 ac	0.74 ± 0.06 b	1.84 ± 0.08 c	2.06 ± 0.23 c	0.99 ± 0.13 ab	0.98 ± 0.04	3.18 ± 2.68	0.90 ± 0.25	1.27 ± 0.16	0.88 ± 0.06
	*Pseudomonas*	1.15 ± 0.24 a	0.15 ± 0.03 b	0.78 ± 0.07 a	0.90 ± 0.22 ac	0.26 ± 0.08 bc	0.06 ± 0.03 a	2.69 ± 2.68 ab	0.21 ± 0.09 ab	0.35 ± 0.18 ab	0.28 ± 0.04 b

*Data is presented as mean ± SEM. Different letters indicate significant differences in relative abundances affected by soil treatments within site. Tukey test, p < 0.05 and n = 3 (R3.2.2). Increased and decreased bacterial relative abundances in treated replant disease (RD) soils compared to untreated within site are highlighted in green and red, respectively. Colored cells indicate those changes that were found at both sites*.

For soil treated with *B. juncea*, no common responders were discovered due to high standard deviations within the treatment (both sites). At site K, members of *Arthrobacter* were the most dominant in soil treated with *B. juncea* (5.89%) and their relative abundances were about three times higher than those in untreated RD soil (Table 3).

Members of the bacterial genus *Arthrobacter* were recorded in significantly enhanced abundance in soils treated with *R. sativus* (8.61 and 4.33% for sites K and A, respectively) compared with untreated RD soils. Another bacterial genus *Terrabacter* was a common responder in soils treated with *R. sativus* being significantly enriched at both sites (Table 3).

For RD soils planted with *Tagetes*, because of site-dependent effects, no common responders were observed for bacteria at the genus levels. A less pronounced effect on the relative abundance of bacterial genera in *Tagetes*-treated soil compared with the other treatments corresponds to the results of the PCoA and the analysis of similarity (Tables 2, 3; Figure 1).

The bacterial genus *Streptomyces* was significantly reduced in relative abundance about 4- to 5-fold after all treatments at site K (Table 3). Irrespective of the soil treatment and the site, Pearson correlation coefficient analysis revealed several bacterial genera to be significantly and positively correlated with growth of apple rootstock M106 plants (SFM or RFM), such as *Arthrobacter, Curtobacterium, Terrimonas, Ferruginibacter* amongst others (Table 4). These bacteria showed higher relative abundances in treated RD soils at site K than at site A (Table 3).

**Table 4:** Pearson correlation coefficient (*r*) between bacterial relative abundance and growth of apple rootstock M106 plants in the field.

**Phylum**	**Genus**	**Relative abundance (%)**	**SFM**		**RFM**	
			***r***	***p*-value**	**r**	***p*-value**
*Actinobacteria*	*Arthrobacter*	3.31 ± 0.45	0.43	0.019	0.25	0.192
	*Curtobacterium*	0.14 ± 0.03	0.46	0.010	0.56	0.001
*Bacteroidetes*	*Terrimonas*	2.46 ± 0.23	0.66	0.000	0.63	0.000
	*Ferruginibacter*	1.11 ± 0.10	0.47	0.009	0.43	0.017
	*Unclass_Flavobacteriaceae*	0.49 ± 0.06	0.50	0.005	0.55	0.002
	*Flavitalea*	0.54 ± 0.08	−0.40	0.028	−0.43	0.018
*Betaproteobacteria*	*Massilia*	0.26 ± 0.04	0.35	0.062	0.45	0.012
*Alphaproteobacteria*	*Sphingomonas*	0.09 ± 0.03	0.29	0.124	0.44	0.015

*Relative abundance is presented as mean ± SEM. SFM, shoot fresh mass and RFM, root fresh mass. The Pearson correlation coefficient was evaluated by Past3 with n = 3*.

### Effects of treatments on soil fungal community composition and diversity

The fungal ITS sequence reads ranged from 24,479 to 34,494 and from 27,123 to 36,234 in soils at sites K and A, respectively, for the different treatments. By trend, higher numbers were displayed in Basamid-treated soils (sites K and A, Table 5). After rarefied sequence data, the OTU numbers and diversity indices were significantly lower in Basamid-treated soil compared to untreated RD soil at site K. At site A, soils treated with *B. juncea* and *R. sativus* possessed significantly more species richness than untreated RD soil. However, the fungal diversity indices were not influenced by any of the treatments in relation to untreated RD soil (Shannon indices, Table 5). Regardless of different soil treatments, the fungal community compositions and diversity were significantly higher in soils at site A than at site K (Table S4; Figure S2).

**Table 5:** Fungal community diversity based on operational taxonomic units (OTUs) at 95% similarity in different soil treatments.

**Site**	**Treatment**	**Sequences per condition**	**Number of OTUs (95%)**	**Chao1**	**Shannon**
K	K_RD	32,718 ± 3,916	112 ± 2 a	130 ± 2	3.13 ± 0.09 a
	K_Basamid	34,494 ± 1,908	86 ± 2 b	121 ± 18	2.36 ± 0.19 b
	K_*B. juncea*	28,665 ± 3,258	105 ± 1ab	120 ± 3	2.72 ± 0.05 ab
	K_*R. sativus*	28,592 ± 3,253	107 ± 3 a	135 ± 10	2.80 ± 0.08 a
	K_*Tagetes*	24,479 ± 5,631	112 ± 10 a	123 ± 14	2.94 ± 0.09 a
A	A_RD	27,123 ± 6,325	119 ± 3 a	126 ± 5 a	2.88 ± 0.18
	A_Basamid	36,234 ± 3,054	117 ± 9 a	132 ± 12 a	2.80 ± 0.20
	A_*B. juncea*	28,425 ± 3,014	151 ± 8 b	179 ± 15 b	3.21 ± 0.09
	A_*R. sativus*	29,545 ± 4,991	151 ± 5 b	175 ± 3 b	3.06 ± 0.09
	A_*Tagetes*	31,643 ± 980	128 ± 10 ab	142 ± 12 a	3.26 ± 0.10

*Data is presented as mean ± SEM. RD, replant disease soil. Letters indicate significant differences within site, Tukey test p < 0.05 and n = 4, except for the RD soil treated with Tagetes, n = 3. Within site, increased and decreased bacterial richness and diversity in treated RD soils compared to untreated are highlighted in green and red, respectively*.

**Figure 3:**
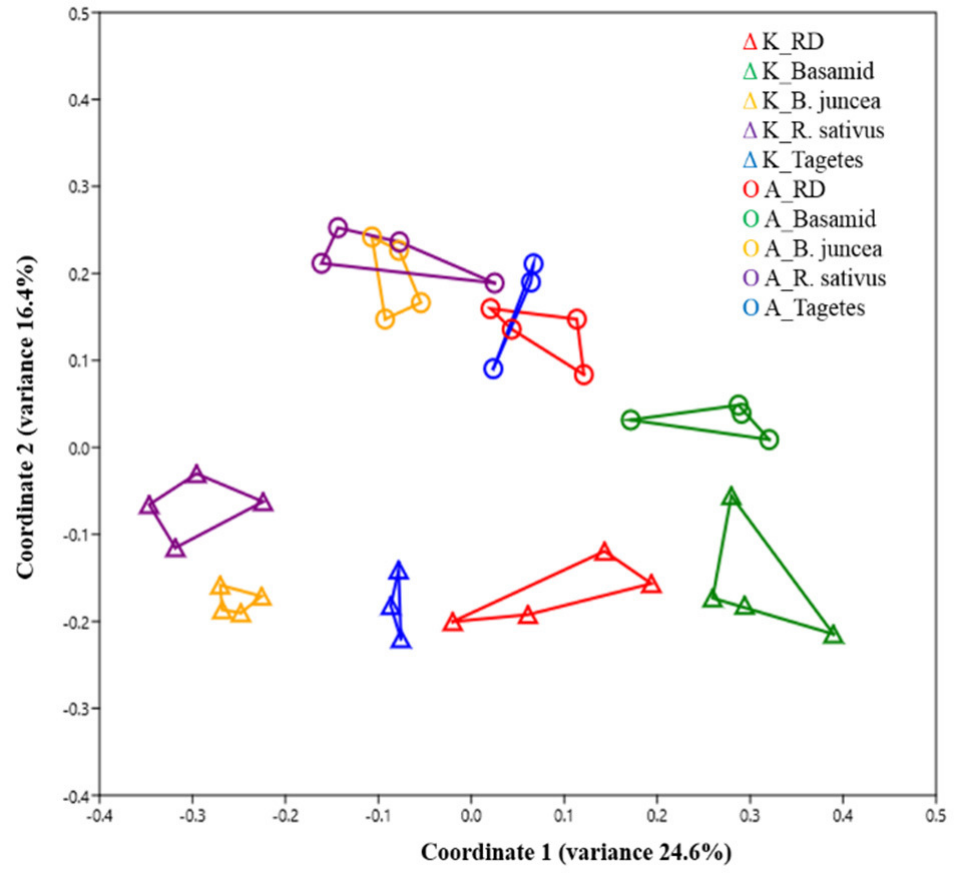
Effect of different treatments on soil fungal community composition under field conditions revealed by principal coordinate analysis (PCoA) using Bray-Curtis distance metric. Past3 with *n* = 4, except for the treatment with *Tagetes, n* = 3. Soil samples were taken 4 weeks after different treatments in September 2013.

As also observed for soil bacteria, differences in fungal community composition between sites were demonstrated (*R* = 0.40 and *p* < 1E-04, Table 6; Figure 3). Effects of the different soil treatments on fungal community composition were clearly stronger compared to effects seen on the bacterial community composition (Tables 2, 6; Figures 1, 3), especially at site K. Significantly different soil fungal community compositions between untreated RD soils and all kinds of treatments were found, except for the soil from *Tagetes* treatment at site A (Table 6).

**Table 6:** Analysis of similarities of the fungal community composition detected in different soil treatments compared with replant disease soil based on OTUs of fungal ITS regions.

**Treatment**	**Site K**		**Site A**	
	***R*-value**	***p*-value**	***R*-value**	***p*-value**
Basamid	0.59	0.030	0.65	0.025
*B. juncea*	1.00	0.031	0.31	0.028
*R. sativus*	1.00	0.028	0.64	0.029
*Tagetes*	0.74	0.030	0.13	0.310

*For sites K vs. A, R-value = 0.40 and p < 0.0001. R- (−1 to 1) and p-values were obtained from ANOSIM-test. R-value close to “1” suggests strong dissimilarity between the communities being compared while an R-value close to “0” represents an even distribution of the communities within and between treatments*.

**Figure 4:**
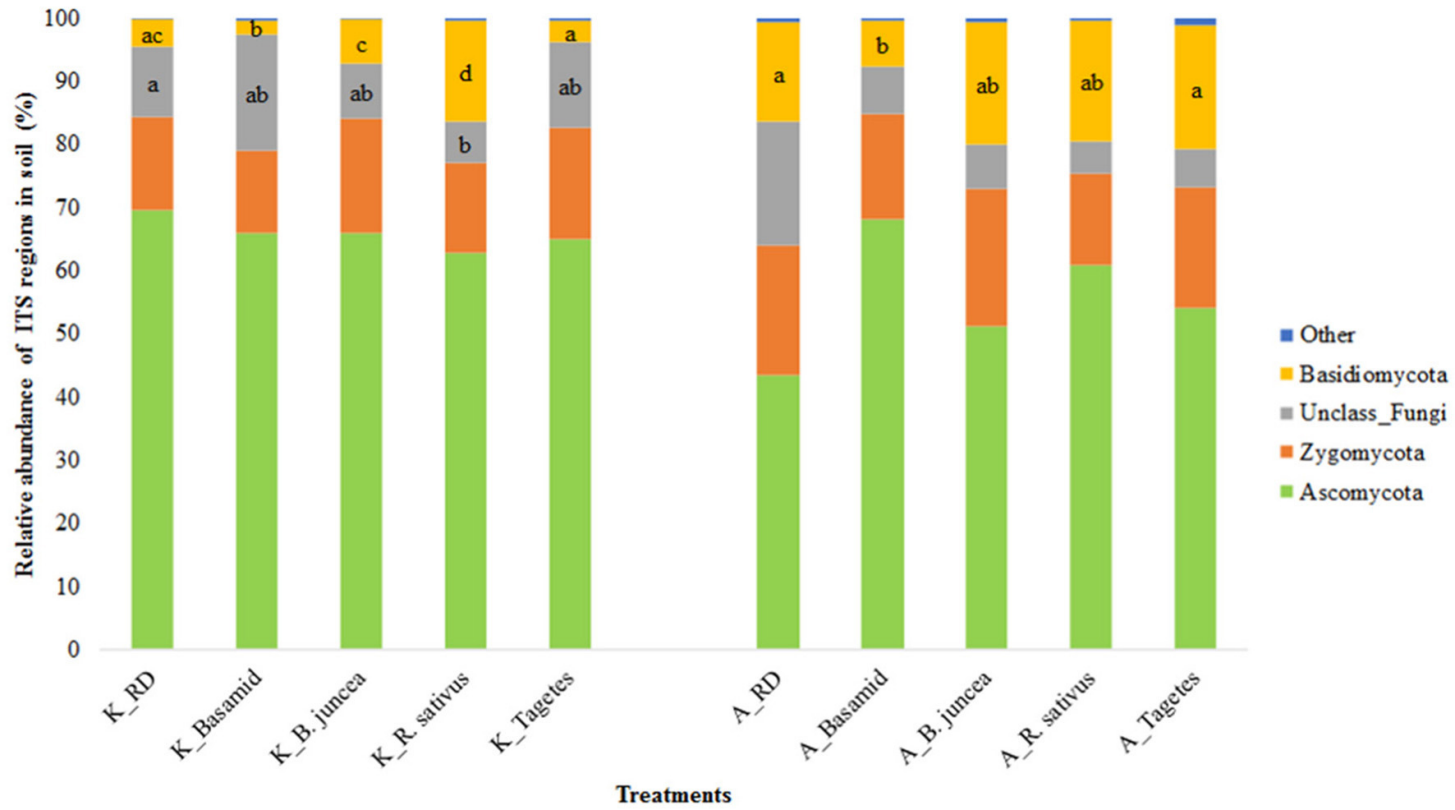
Relative abundance of dominant fungal phyla in soils at the two sites affected by the different treatments. Different letters within the phylum indicate significant differences between soil treatments within site, Tukey test, *p* < 0.05 and *n* = 4, except for the soil treated with *Tagetes, n* = 3.

The fungal phylum *Ascomycota* was most abundant in all soils and at all sites (Figure 4; Table S5). Relatively high proportion was observed for unclassified fungi, accounting for 11.03 and 19.43% in RD soils at sites K and A, respectively (Figure 4). The fungal phylum *Basidiomycota* was significantly reduced in relative abundance by about 50% after Basamid treatment at both sites. Its members were found significantly increased (3.7-fold) by the *R. sativus* treatment at site K, but not significantly at site A. Here, high variations among the replicates were recorded and no significant effects of the treatments were detected, except for those mentioned for *Basidiomycota* (Figure 4).

Due to the high standard deviations, only fungal sequences affiliated to *Leotiomycetes (Incertae sedis)*, were identified as common responder to the Basamid treatment with significantly higher relative abundance compared to untreated RD soils (Table 7). Similar responses in RD soil biofumigated with either *B. juncea* or *R. sativus* were obtained for the fungal genera *Podospora, Monographella*, and *Mucor*, all of them significantly increasing in relative abundance, and for *Ypsilina*, the proportions of which significantly decreased at both sites. Among them, the fungal genera *Podospora* (19.19%) and *Monographella* (16.52%) had the highest relative abundances in soil treatments with *B. juncea* at site K and *R. sativus* at site A, respectively (Table 7). Regarding soils treated with *Tagetes*, more pronounced effects were observed at site K than at site A. Not only the analysis of similarity showed a significant higher *R*-value (0.74), but also several fungal genera were highly affected in their population compared to the untreated RD soil, e.g., members of unclassified *Pleosporales, Tetracladium* and unclassified *Sordariomycetes* (site K, Tables 6, 7).

**Table 7:** Relative abundance of fungal genera detected in TC-DNAs extracted from bulk soils taken 4 weeks after different treatments at two replant disease sites (only genera with a relative abundance > 0.5 % are shown).

**Phylum/ Family**	**Genus**	**Site K**					**Site A**				
		**K_RD**	**K_Basamid**	**K_*B. juncea***	**K_*R. sativus***	**K_*Tagetes***	**A_RD**	**A_Basamid**	**A_*B. juncea***	**A_*R. sativus***	**A_*Tagetes***
***Ascomycota***											
*Unclass_Pleosporales*	*Unclass_Pleosporales*	6.35 ± 0.78a	3.44 ± 0.67ac	1.17 ± 0.08 b	0.80 ± 0.07 b	2.10 ± 0.24 c	5.22 ± 1.46	3.36 ± 1.20	4.51 ± 0.78	2.91 ± 0.39	5.25 ± 1.27
*Pleosporaceae*		0.48 ± 0.13	0.11 ± 0.05	0.55 ± 0.22	0.16 ± 0.03	1.18 ± 0.45	0.29 ± 0.11ab	0.06 ± 0.04a	0.83 ± 0.20b	0.37 ± 0.07b	0.45 ± 0.11ab
	*Dendryphion*	0.09 ± 0.02a	0.00 ± 0.00 b	0.41 ± 0.20abc	0.11 ± 0.01a	1.14 ± 0.46 c	0.20 ± 0.10ab	0.02 ± 0.01a	0.60 ± 0.21b	0.22 ± 0.07b	0.28 ± 0.08b
*Trichocomaceae*		0.47 ± 0.08a	6.82 ± 2.05 b	0.77 ± 0.22a	1.84 ± 1.14ab	0.28 ± 0.01a	3.46 ± 1.10ab	5.24 ± 0.90b	1.89 ± 0.22a	4.61 ± 1.63ab	1.59 ± 0.15a
	*Penicillium*	0.34 ± 0.10a	6.67 ± 2.05 b	0.70 ± 0.19a	1.76 ± 1.15ab	0.21 ± 0.05a	3.15 ± 1.10ab	3.59 ± 0.45a	1.23 ± 0.23b	4.23 ± 1.51ab	0.96 ± 0.12b
*Incert_sed_Ascomycota*		2.29 ± 0.57a	0.36 ± 0.24 b	0.23 ± 0.07 b	0.37 ± 0.06 b	2.82 ± 0.39a	1.68 ± 0.48ac	0.20 ± 0.08 b	0.44 ± 0.07ab	0.56 ± 0.13bc	0.84 ± 0.07c
	*Ypsilina*	1.61 ± 0.38ab	0.30 ± 0.24bc	0.04 ± 0.03 c	0.07 ± 0.01 c	2.29 ± 0.38a	1.49 ± 0.39a	0.07 ± 0.04 b	0.16 ± 0.01 b	0.18 ± 0.01 b	0.74 ± 0.03a
*Incert_sed_Helotiales*		5.12 ± 0.53a	0.49 ± 0.37 b	0.57 ± 0.15 b	0.72 ± 0.14 b	10.96 ± 0.92 c	1.69 ± 0.65ab	1.15 ± 0.64ab	0.54 ± 0.17b	0.36 ± 0.14b	2.94 ± 0.48a
	*Tetracladium*	4.12 ± 0.73a	0.09 ± 0.05 b	0.44 ± 0.11 bc	0.67 ± 0.13 c	10.41 ± 0.99 d	1.08 ± 0.34ab	0.84 ± 0.63ab	0.29 ± 0.10a	0.28 ± 0.10a	2.66 ± 0.43b
*Incert_sed_Leotiomycetes*		0.39 ± 0.12a	4.17 ± 1.17 b	0.09 ± 0.04a	0.11 ± 0.04a	0.22 ± 0.04a	0.42 ± 0.07a	5.61 ± 1.94 b	0.30 ± 0.07a	0.20 ± 0.04a	0.38 ± 0.09a
	*Incert_sed_Leotiomycetes*	0.17 ± 0.04a	3.88 ± 1.01 b	0.04 ± 0.02a	0.10 ± 0.03a	0.06 ± 0.02a	0.41 ± 0.06a	5.42 ± 1.96 b	0.22 ± 0.03a	0.19 ± 0.04a	0.23 ± 0.12a
*Myxotrichaceae*	*Pseudogymnoascus*	6.02 ± 2.13ab	26.19 ± 10.85b	0.62 ± 0.14 cd	0.38 ± 0.06 c	1.20 ± 0.13ad	0.58 ± 0.15a	10.85 ± 3.16 b	0.47 ± 0.31a	0.54 ± 0.28a	0.33 ± 0.17a
*Ascobolaceae*		5.68 ± 2.02ac	0.09 ± 0.07 b	8.87 ± 2.12ac	16.83 ± 5.59c	2.46 ± 0.53a	0.58 ± 0.26	0.09 ± 0.06	0.39 ± 0.18	1.09 ± 0.49	1.20 ± 0.64
	*Ascobolus*	5.68 ± 2.02ac	0.09 ± 0.07 b	8.86 ± 2.11ac	16.83 ± 5.59c	2.44 ± 0.53a	0.55 ± 0.26	0.01 ± 0.00	0.37 ± 0.17	1.04 ± 0.50	1.13 ± 0.58
*Unclass_Sordariomycetes*	*Unclass_Sordariomycetes*	2.09 ± 0.87a	1.09 ± 0.12a	11.64 ± 1.78 b	15.06 ± 0.38 b	16.15 ± 5.84 b	3.19 ± 0.95	3.17 ± 1.02	5.08 ± 1.32	4.52 ± 0.84	3.28 ± 1.29
*Unclass_Sordariales*	*Unclass_Sordariales*	1.51 ± 0.40a	0.82 ± 0.28a	6.99 ± 1.14 b	1.12 ± 0.19a	3.16 ± 1.21ab	0.52 ± 0.08a	0.63 ± 0.11a	1.35 ± 0.28ab	1.54 ± 0.68ab	2.21 ± 0.48 b
*Chaetomiaceae*		0.69 ± 0.06ab	0.36 ± 0.10a	0.47 ± 0.10a	1.30 ± 0.21b	0.53 ± 0.13ab	0.36 ± 0.09ab	0.19 ± 0.06a	1.11 ± 0.24b	1.12 ± 0.22b	0.51 ± 0.11ab
	*Unclass_Chaetomiaceae*	0.28 ± 0.06a	0.03 ± 0.00 b	0.36 ± 0.09a	1.19 ± 0.21 c	0.31 ± 0.06a	0.33 ± 0.09a	0.15 ± 0.05a	1.00 ± 0.17 b	1.05 ± 0.20 b	0.42 ± 0.06ab
*Lasiosphaeriaceae*		0.50 ± 0.19a	0.84 ± 0.28a	20.02 ± 1.23 b	6.19 ± 0.52c	1.56 ± 0.36a	0.25 ± 0.06a	0.54 ± 0.15ab	2.16 ± 0.55 b	1.51 ± 0.60ab	1.21 ± 0.23 b
	*Podospora*	0.20 ± 0.12a	0.22 ± 0.12a	19.19 ± 1.06 b	5.59 ± 0.45 c	0.19 ± 0.04a	0.02 ± 0.01a	0.04 ± 0.01a	1.48 ± 0.56 b	0.39 ± 0.07 b	0.01 ± 0.01a
*Incert_sed_Xylariales*	*Monographella*	0.53 ± 0.26a	0.11 ± 0.02a	2.56 ± 0.37 bc	4.21 ± 0.53 c	1.92 ± 0.21 b	0.22 ± 0.10a	0.13 ± 0.03a	7.47 ± 1.08 b	16.52 ± 4.46 b	0.60 ± 0.24a
***Basidiomycota***											
*Incert_sed_Tremellales*		2.20 ± 0.61ab	1.00 ± 0.29a	2.91 ± 0.41b	7.72 ± 0.76 c	2.00 ± 0.28ab	11.44 ± 1.75ac	2.60 ± 0.58 b	10.10 ± 0.61a	12.64 ± 2.70ac	14.58 ± 0.10c
	*Cryptococcus*	2.17 ± 0.59ab	0.85 ± 0.27a	2.79 ± 0.40b	7.63 ± 0.76 c	1.93 ± 0.28ab	11.28 ± 1.72ac	2.49 ± 0.60 b	9.89 ± 0.60a	12.51 ± 2.70ac	14.30 ± 0.17c
*Trichosporonaceae*	*Trichosporon*	0.18 ± 0.07a	0.15 ± 0.09a	3.39 ± 0.84b	6.43 ± 1.83 b	0.06 ± 0.01a	0.66 ± 0.26a	0.21 ± 0.09a	7.61 ± 4.31ab	4.93 ± 0.49 b	0.35 ± 0.13a
***Zygomycota***											
*Mucoraceae*	*Mucor*	0.30 ± 0.05a	0.16 ± 0.08a	0.85 ± 0.06 b	2.00 ± 0.37 c	1.00 ± 0.68*abc*	0.47 ± 0.17a	1.12 ± 0.21ab	3.33 ± 0.95 b	2.89 ± 0.50 b	0.61 ± 0.16a

*Data is presented as mean ± SEM. Different letters indicate significant differences in relative abundances affected by soil treatments within site. Tukey test, p < 0.05 and n = 4, except the soil treated with Tagetes, n = 3 (R3.2.2). Increased and decreased fungal relative abundances in treated replant disease (RD) soils compared to untreated within site are highlighted in green and red, respectively. Colored cells indicate those changes that were found at both sites*.

Irrespective of soil treatments and sites, members of unclassified *Pleosporales, Cryptococcus*, and *Mucor* were negatively and significantly correlated with growth of apple rootstock M106 plants (shoot and root). Correspondingly, the relative abundance of unclassified *Pleosporales* was significantly reduced after treatments with *B. juncea, R. sativus*, and *Tagetes* at site K (Tables 7, 8). The remarkably increased relative abundance of members of unclassified *Sordariomycetes* in *B. juncea* (11.64%), *R. sativus* (15.06%), and *Tagetes* (16.15%) soils at site K were positively and significantly correlated with the growth of M106 plants. Furthermore, a positive correlation to growth of the apple M106 plants was demonstrated for the fungal genera *Podospora* and unclassified *Sordariales* (Table 8).

**Table 8:** Pearson correlation coefficient (r) between fungal relative abundance and growth of apple rootstock M106 plants in the field.

**Phylum**	**Genus**	**Relative abundance (%)**	**SFM**		**RFM**	
			***r***	***p*-value**	***r***	***p*-value**
*Ascomycota*	*Unclass_Pleosporales*	3.58 ± 0.43	−0.57	0.001	−0.37	0.044
	*Unclass_Sordariomycetes*	6.57 ± 1.11	0.54	0.002	0.39	0.035
	*Unclass_Sordariales*	1.98 ± 0.39	0.44	0.016	0.23	0.218
	*Podospora*	2.76 ± 1.08	0.38	0.036	0.17	0.364
*Basidiomycota*	*Cryptococcus*	6.54 ± 0.99	−0.36	0.049	−0.54	0.002
*Zygomycota*	*Mucor*	1.26 ± 0.23	−0.22	0.239	−0.40	0.027

*Relative abundance is presented as mean ± SEM. SFM, shoot fresh mass and RFM, root fresh mass. Past3 and n = 4, except for the treatment with Tagetes, n = 3*.

## Discussion

Changes in bacterial and fungal community composition and relative abundances based on Illumina sequencing of 16S rRNA gene or ITS fragments amplified from TC-DNAs extracted from soils after treatments with Basamid, *B. juncea, R. sativus*, and *Tagetes* were investigated via comparison to corresponding untreated RD soils at two sites in order to identify causes for the differentially improved plant growth in treated soils.

The observed differences in soil bacterial and fungal community compositions between the two RD sites were in line with our previous findings (Yim et al., 2015, 2016). The two RD sites differed in soil type, soil physical and chemical properties and soil cultivation and management history (Yim et al., 2015, 2016). Different soil microbiomes with different capacities in RD development of the two studied sites were in line with previous observations of soil microbiomes being shaped by different plant species or genotypes (St. Laurent et al., 2010; Uroz et al., 2016), soil types and soil amendments like mineral nutrients (Bakker et al., 2015).

Also the soil treatments differed in their efficacy in a site dependent way (Figures 1, 3; Tables 3, 7). This is most likely due to the fact that ITCs, the toxic compounds released from the treatments with Basamid (methyl-ITC), *B. juncea* (allyl-ITC) and *R. sativus* (4-methylthio-3-butenyl-ITC) differed in their profiles and concentrations depending on the site (Yim et al., 2016). Variations in toxicity of different ITC compounds against tested pathogens were previously reported (Neubauer et al., 2014).

The analyzed samples were taken 4 weeks after different treatments (*B. juncea, R. sativus*, and Basamid). Thus, changes in relative abundance of bacteria and fungi in treated soils with *B. juncea* and *R. sativus* can possibly be explained with the effects of plant root exudation (Bertin et al., 2003; Berg and Smalla, 2009; Schreiter et al., 2014), toxicity of ITCs released from the treatments (Neubauer et al., 2014; Hanschen et al., 2015), a huge amount of plant biomass incorporation into treated soils as well as nutrients released from plant biomass degradation as previously reported (Bakker et al., 2015; Yim et al., 2016). Flavonoids and other phenolic compounds were also reported to be present in Brassicaceae tissues (Antonious et al., 2009; Cartea et al., 2011) and were shown to influence the soil microbiome (Weston and Mathesius, 2013). Analyses with samples taken at different time points could resolve responders that were affected by those different effects. Regarding the Basamid treatments, altering soil bacterial and fungal relative abundances possibly resulted from combinations of a direct toxic effect of methyl-ITC released from the treatment, recolonization and niche competition of taxa recovering from the treatments (Ridge and Theodorou, 1972; Neumann et al., 1983; Hibbing et al., 2010).

Microbial taxa associated with apple RD symptoms were not consistently detected in the recent TC-DNAs based studies in apple RD soils (Sun et al., 2014; Franke-Whittle et al., 2015; Yim et al., 2015; Nicola et al., 2017). For example, several bacterial genera such as *Gp5, Gp6, Gp9, Geobacter* (Nicola et al., 2017), *Gemmatimonas, Devosia, Sphingomonas* (Franke-Whittle et al., 2015), *Phenylobacterium* and *Lysobacter* (Sun et al., 2014; Franke-Whittle et al., 2015) and the fungal genera *Cryptococcus, Mortierella*, and *Tricharina* (Nicola et al., 2017) were not commonly identified to be linked with apple RD incidence among studies in which their relative abundances were negatively correlated with growth of apple plants. In the present study, the bacterial genus *Flavitalea* and the fungal genera unclassified *Pleosporales, Cryptococcus*, and *Mucor* could be associated with RD incidence with M106 plants as indicated by a negative correlation to the shoot or root growth (Tables 4, 8). In contrast, the bacterial genera *Arthrobacter, Curtobacterium, Terrimonas, Ferruginibacter* and the fungal genera unclassified *Sordariomycetes*, unclassified *Sordariales* and *Podospora* revealed a positive correlation to the shoot or root growth of M106 plants.

The positive and negative correlations of the fungal genera *Podospora* and *Cryptococcus*, respectively, to plant growth in the present study were in agreement with the observations by Franke-Whittle et al. (2015) who analyzed microbial communities at different apple replant disease sites. The relative abundances of several bacterial genera, like *Arthrobacter, Terrimonas*, and *Ferruginibacter* and fungal genera, for instance *Podospora* that were positively and significantly correlated with growth of the apple M106 plants (Tables 4, 8) were lower in RD soils treated with Basamid, *B. juncea, R. sativus*, and *Tagetes* at site A than at site K (Tables 3, 7). These differences might contribute to explain the lower effectiveness of these treatments at site A revealed by the growth of M106 plants. Thus, knowing RD site specificities such as its local selected microbiomes influenced by soil properties, soil quality, and pedoclimatic conditions is an important point before choosing the right RD management strategies. Such sequence approaches used in the present work are important in identifying potential bioindicators in the RD soils (Nunes et al., 2016; Schöler et al., 2017).

The effects of the *Tagetes* treatment on soil bacterial and fungal community composition (Tables 2, 6; Figures 1, 3) and relative abundances of different fungal and bacterial genera (Tables 3, 7) were lower than those resulting from *B. juncea* and *R. sativus* treatments. This might at least partially be due to the fact that samples were taken when *Tagetes* plants were still growing in 2013, thus only root exudates, but not plowed plant biomass could contribute to the observed effects. Shifts in bacterial and fungal relative abundances in the *Tagetes*-treated soils would probably have been higher if the analyzed samples had been taken 4 weeks after plant tissue incorporation. In 2012, however, the total plant biomass from *Tagetes* was incorporated into the soil. Therefore, several bacterial and fungal groups were significantly altered in abundance by this treatment, although site-dependently (Tables 3, 7). *Tagetes* are known as nematode-repellent plants due to their sulfur-containing heterocyclic compounds, thiophenes, produced by plant roots (Marotti et al., 2010; Marahatta et al., 2012; Saha et al., 2012). In the present study, soil-borne plant endoparasitic nematode *Pratylenchus* sp. which has previously been reported to be associated with apple RD soil (Mai et al., 1994) was strongly reduced in *Tagetes-*treated soil compared with the untreated RD soils, especially at site A (Table S6). Besides thiophenes, terpenoids including dihydrotagetone, piperitone and α-terpineol were predominantly identified in leaves and flowers of *Tagetes* (Saha et al., 2012). The thiophenes and terpenoids showed highly suppressive potential for several soil-borne and foliar plant pathogenic fungi of several crops such as finger millet (*Pyricularia grisea*), French bean (*R. solani, F. solani*, and *Sclerotium rolfsii*), pea (*Fusarium oxysporum*), and tomato (*Alternaria solani*) in an *in vitro* study (Saha et al., 2012). Despite the less pronounced changes in soil bacterial and fungal community composition in soils cropped with *Tagetes* plants compared to other treatments (Tables 3, 7; Figures 1, 3), interestingly, the growth of the indicator plants, M106, showed comparable effects among all treatments at site K (Table S1). Therefore, soil-borne pathogenic nematodes were possibly one of the causal ARD agents in the analyzed soils that were suppressed by the *Tagetes* treatment.

The stronger effect observed on fungal community compositions in RD soils treated with *B. juncea* and *R. sativus* compared to bacteria (Figures 1, 3; Tables 2, 6) confirmed the observations made in several other studies when the soils were submitted to products containing ITCs (Hollister et al., 2013; Hu et al., 2015). Interestingly, at site K, a higher effect on soil fungi and a lower effect on soil bacteria in RD soils treated with *B. juncea, R. sativus*, and *Tagetes* (*R*-values, Tables 2, 6) was found in line with the biomass of apple rootstock M106 plants being significantly higher only at this site as well (Table S1; Yim et al., 2016). This shows that soil at site K was more affected by RD, pointing to a more important role of fungi in RD incidences, as stated earlier by Mazzola (1998).

### Bacterial responders to the different treatments of replant disease soils

A pronounced and significant enrichment of the bacterial phylum *Actinobacteria* was observed in RD soils treated with *R. sativus* at sites K and A (Figure 2; Table S3). Many members of this phylum are known as plant growth promoting (PGP) bacteria being involved in soil-borne disease suppression (Palaniyandi et al., 2013). A closer look at the genus levels of the responders belonging to this phylum revealed that *Arthrobacter* shared the highest proportion in the RD soils when they had been treated with *B. juncea* (at site K) or *R. sativus* (at both sites) (Table 3). *Arthrobacter* sp. was previously reported as PGP bacterium, as degrader of phenolic compounds in soil (Karigar et al., 2006; Unell et al., 2008) and releasing plant-available iron (Valencia-Cantero et al., 2007). Siddikee et al. (2010) identified traits of isolates affiliated to *Arthrobacter nicotianae* such as nitrogen fixation, indole acetic acid (IAA) production to promote root growth of plants, thiosulfate oxidation, ammonia production and 1-aminocyclopropane-1-carboxylic acid (ACC) deaminase activity strengthening plants to tolerate salt stress conditions. The bacterial genus *Arthrobacter* was also significantly higher in relative abundance in RD soils treated with gamma irradiation and concomitantly, apple plant growth was significantly enhanced in irradiated soils (Yim et al., 2015). Hence, *Arthrobacter* species in biofumigated soils possibly contributed to enhanced growth of M106 plants.

Furthermore, other members of *Actinobacteria* such as *Salinibacterium* and *Curtobacterium* also responded to the Basamid treatments at sites K and A (Table 3). These bacterial groups were possibly involved in biodegradation of the Basamid remnant in the soil. The *Curtobacterium* sp. strain 114-2 was capable to degrade the toxic trichothecenes in culture medium (Ueno et al., 1983). Moreover, *Curtobacterium flaccumfaciens* strain ME1 was discovered to promote the plant growth and to protect cucumber plants from leaf spot disease (Raupach and Kloepper, 2000). In addition, this strain was reported to have an effect comparable to the soil fumigant methyl bromide (Raupach and Kloepper, 2000). Other plant growth promoting traits such as solubilizing phosphate, producing IAA as well as catalase and ACC deaminase activity were reported for the *Curtobacterium* sp. strain S6 (Bulgari et al., 2014). Therefore, increased relative abundance of *Curtobacterium* in Basamid treated soils might point to species that promoted growth of M106 plants.

Members of the bacterial genus *Ferruginibacter* (phylum *Bacteroidetes*) which were identified in significantly higher abundance in *B. juncea* (site K) and *R. sativus* (site A) treated soils compared with untreated RD soil (Table 3) were demonstrated to be able to decompose cellulose (Lewin et al., 2016). Cellulose is the major component of cell walls of plants (Kögel-Knabner, 2002) and oomycetes (Mélida et al., 2013). Therefore, it cannot be excluded that these members (*Ferruginibacter*) play a role in carbon mineralization and oomycete cell wall degradation in the treated soil. The genera *Pythium* (Hoestra, 1994; Emmett et al., 2014) and *Phytophthora* (Mazzola, 1998; Tewoldemedhin et al., 2011; Kelderer et al., 2012) belonging to the oomycetes were previously reported to be associated with apple RD incidence. Thus, for instance *Ferruginibacter* which was detected in higher relative abundance in soils treated with *B. juncea* (site K) and *R. sativus* (site A) might have antagonistic activity against apple plant pathogenic oomycetes in the present study.

The enrichment of the genus *Rhodanobacter* in Basamid soil at sites K and A was in line with its detection in higher abundance in gamma-irradiated RD soil (Yim et al., 2015), and the apple plants were significantly increased in their biomass in this treated soil.

The significant increase in *Massilia* relative abundance in Basamid soil at site K and its positive correlation with plant growth (Tables 3, 4) suggest that it might be part of a beneficial soil bacterial group, as this genus contains species that are able to produce and secrete chitinase (Cretoiu et al., 2013). Activating chitin degraders in soils has been shown to be related with the suppression of plant pathogens containing chitin structures like fungal cell walls and the exoskeleton of invertebrates (Rinaudo, 2008; Hjort et al., 2009; Jacquiod et al., 2013). The bacterial genus *Massilia* was also reported to show a positive correlation to the shoot growth of apple plants grown in ARD soils in a recent TC-DNA based study (Nicola et al., 2017).

Although members of the genus *Pseudomonas* were significantly reduced in relative abundance in soils treated with Basamid and *Tagetes* at site K, their abundances were not negatively associated with the growth of apple M106 plants in the present investigation (Table 3). *Pseudomonas* sp. is known as a beneficial bacterium for plant growth since it enhances sulfate uptake (Behera et al., 2014) and acts as antagonist against soil pathogenic fungi (Zaccardelli et al., 2013). At the same time, the genus contains plant pathogens; therefore, an identification of the species would be needed to enable statements on their effects. A significantly decreased relative abundance of *Streptomyces* in all treated soils at site K and an increase of relative abundances of *Arthrobacter* in *B. juncea* (site K) and *R. sativus* (sites K, A) soils observed in the present study was also reported by Mazzola et al. (2015) when soils were treated with seed meal from *Brassica* crops.

### Fungal responders to the different treatments of replant disease soils

In the present study, a huge amount of plant biomass from *B. juncea* and *R. sativus* was incorporated into soils for biofumigation, and thus enhanced fungal groups that are potentially able to degrade plant celluloses were recorded. Among identified responders, cellulose degraders were previously reported for isolates belonging to the fungal genera *Trichosporon* (Santos and Linardi, 2001; Štursová et al., 2012), *Mucor* (Mahmood et al., 2006), and *Podospora* (Couturier et al., 2016).

The fungal genus *Podospora* contains *Podospora anserina* as a coprophilous fungus which is efficient in degrading plant biomass due to its lignocellolytic enzymes (Couturier et al., 2016). Besides, the genus *Podospora* was also previously shown to enhance root growth of pea plants (Xu et al., 2012). Moreover, the positive correlation of the fungal genus *Podospora* to apple growth was also recorded by Franke-Whittle et al. (2015). Thus, the significantly increased relative abundance of *Podospora* in *B. juncea* and *R. sativus* treated soils at both sites in the present study (Table 7) might suggest that these taxa contributed to antagonism relationship with pathogenic microorganisms in apple RD soils.

A high relative abundance in soils treated with *B. juncea* or *R. sativus* (at both sites) and planted with *Tagetes* at site K was also recorded for the fungal genus *Monographella* (Table 7). Berg et al. (2005) reported that isolates of the genus *Monographella* from the rhizosphere of *Brassica napus* plants displayed antagonistic activity against *Verticillium dahliae* Kleb.

The significantly enriched members of *Penicillium* in Basamid-treated soil (site K) and *Trichosporon* in *B. juncea-* (site K) and *R. sativus-* (sites K, A) treated soils were in agreement with the study of Franke-Whittle et al. (2015) who assumed these genera to be beneficial for growth of apple rootstock plantlets.

Members of *Tetracladium* were significantly reduced by treatments with Basamid, *B. juncea* and *R. sativus* at site K (Table 7), which is in contrast to the finding that this fungal group was earlier shown to have a positive effect on growth of apple plants (Franke-Whittle et al., 2015). On the other hand, the relative abundance of members of *Tetracladium* was 2.5 times higher after *Tagetes* treatment than in untreated RD soils at site K (Table 7).

The unclassified fungal genus *Pleosporales* was recorded in a relatively high proportion in untreated RD soils (both sites), but significantly decreased in relative abundance after treatments with *B. juncea, R. sativus*, and *Tagetes* at site K (Tables 7, 8). They are belonging to the order *Pleosporales* which contains several plant pathogens (Zhang et al., 2009). The genome analysis confirmed that the fungal order *Pleosporales* contained several enzymes that are associated with plant pathogenicity (Ohm et al., 2012) such as glycoside hydrolases, lipases and peptidases as well as small secreted protein to infect the plant cells. In the present study, the detected relative abundance of the unclassified *Pleosporales* was negatively correlated with the growth of the apple M106 plants (Table 8). Thus, the suppression of their relative abundance in *B. juncea-, R. sativus-*, and *Tagetes-*treated soils (site K, Table 7) might have positive effects on the plant growth due to possible reduction of specific microbial pathogenic groups. No obvious correlation between bacteria and fungi at the alpha and beta diversity levels could be detected (data not shown). The relative abundance of the fungal unclassified *Pleosporales* in the untreated RD soils was observed to be negatively correlated to several bacterial groups that were significantly enhanced in their relative abundances by the soil treatments (Figure S3). Thus, the interaction between different bacterial and fungal taxa should be studied in detail in further analyses.

The pathogenic oomycetes associated with apple RD incidence such as *Pythium* sp. (Hoestra, 1994; Emmett et al., 2014) and *Phytophthora* sp. (Mazzola, 1998; Tewoldemedhin et al., 2011; Kelderer et al., 2012) were not detected in the present study due to the primer system used. Thus, primers specific for the oomycetes (Riit et al., 2016), should be included for future amplicon studies as well. For future studies, selected bacterial and fungal genera, which were positively and negatively correlated with the growth of the apple plants in the present work should be further investigated and isolated for their potential application in overcoming RD as promising microbial bioindicators in order to better refine our treatment procedures against RD affected soils.

## Conclusion

Bacterial or fungal responders to the soil treatments applied in this study were treatment- and site-dependent. Most importantly, pre-RD soil treatments improved apple growth as previously published (Yim et al., 2016). The positive and significant effects of the different RD soil treatments on growth of the M106 plants at site K were associated with alterations of both bacterial and fungal communities in the treated RD soils. Since more significant changes involved increased abundances of the respective genera, a certain number of beneficial bacterial and fungal genera is possibly required to enhance the plant growth and to counteract plant-pathogens. The enriched bacterial and fungal groups detected should be further studied with regard to their potential roles in overcoming RD. The negative correlation with growth of the M106 plants as well as the high relative abundance of the fungal order *Pleosporales* in the untreated RD soils was possibly an indication of a potential fungal pathogenic group in the analyzed soils. Overall, the present study revealed shifts in the bacterial and even more pronounced in the fungal communities in response to the treatments of RD soils, and the relative abundances of numerous taxa that were positively correlated to apple plant growth were identified.

## Author contributions

BY: Implementing the project; sampling soil from the field, TC-DNA extraction, analyzing and interpreting data of the work and writing the manuscript (MS). HN: Contributing in the project experimental design, providing the nematode data and contributing to improve the MS. AW: Contributing in the project experimental design, soil sampling and performing the field experiment. SJ: Performing Miseq Illumina sequencing, data analyzing and contributing in writing the MS and final approval of the version to be published. SS: Contributing in writing the MS and final approval of the version to be published. TW: Substantial contributions to the conception or design of the work, interpretation of data, writing the MS and final approval of the version to be published. As well as be accountable for all aspects of the work in ensuring that questions related to the accuracy or integrity of any part of the work are appropriately investigated and resolved. KS: Substantial contributions to the conception or design of the work, interpretation of data, writing the MS and final approval of the published version.

### Conflict of interest statement

The authors declare that the research was conducted in the absence of any commercial or financial relationships that could be construed as a potential conflict of interest.
