# Supplementary material for: Effects of Soil Pre-Treatment with Basamid® Granules, Brassica juncea, Raphanus sativus, and Tagetes patula on Bacterial and Fungal Communities at Two Apple Replant Disease Sites
Source: Front Microbiol. 2017 Sep 1;8:1604. doi: 10.3389/fmicb.2017.01604 (PMC5586068; doi:10.3389/fmicb.2017.01604)
Supplement: Supplementary file 1 [file DataSheet1.docx]

**List of appendix**

**List of supplementary tables**

**Table S1: Increases (%) in shoot fresh mass (SFM) of apple rootstock M106 plants in treated soils compared to the mass in replant disease soil**

| **Treatment** | **SFM** | |
| --- | --- | --- |
|  | **Site K** | **Site A** |
| RD | 0 | 0 |
| Basamid | 155*** | 16 |
| *B. juncea* | 148*** | 1 |
| *R. sativus* | 165*** | 10 |
| *Tagetes* | 175*** | 52* |

Evaluation was performed with SFM of M106 plants grown under field conditions in 2014. Significant codes: *, *p* < 0.05; **, *p* < 0.001 and ***, *p* < 0.0001. *LSD test* and n = 3.

**Table S2: Bacterial community diversity based on operational taxonomic units (OTUs) at 97% similarity in soils at two sites**

| **Site** | **Number OTU** | **Chao1** | **Shannon** |
| --- | --- | --- | --- |
| **K** | 334±5 a | 373±6 a | 4.25±0.04 a |
| **A** | 299±9 b | 343±10 b | 3.78±0.13 b |

Data is presented as mean±SEM. Letters indicate significant differences, *Tukey test*, *p* < 0.05 and n = 15. Chao1, species richness. Decreased bacterial richness and diversity is highlighted in red.

**Table S3: Relative abundance of bacterial phyla in soils at two sites affected by different treatments**

| **Phylum** | **Site K** | | | | | **Site A** | | | | |
| --- | --- | --- | --- | --- | --- | --- | --- | --- | --- | --- |
|  | **K_RD** | **K_Basamid** | **K_*B. juncea*** | **K_*R. sativus*** | **K_*Tagetes*** | **A_RD** | **A_Basamid** | **A_*B. juncea*** | **A_*R. sativus*** | **A_*Tagetes*** |
| *Acidobacteria* | 0.03±0.02 | 0.03±0.01 | 0.04±0.02 | 0.03±0.00 | 0.04±0.01 | 0.10±0.01 a | 0.11±0.07 ab | 0.20±0.08 ab | 0.27±0.03 b | 0.10±0.02 a |
| *Actinobacteria* | 13.47±0.77 a | 18.01±1.97 ab | 18.22±1.43 ab | 21.13±0.98 b | 13.55±1.39 a | 10.51±0.52 a | 13.15±0.84 ab | 11.64±3.05 ab | 15.74±0.88 b | 12.65±0.41 ab |
| *Bacteroidetes* | 8.38±0.36 a | 10.67±0.88 ab | 12.79±1.16 b | 11.14±0.27 b | 10.99±1.34 ab | 3.77±0.14 a | 6.70±0.36 b | 9.08±2.71 abc | 10.93±0.16 c | 4.54±0.27 a |
| *Chloroflexi* | 2.19±0.40 | 1.93±0.36 | 2.05±0.17 | 1.98±0.17 | 2.31±0.09 | 1.25±0.11 ab | 0.82±0.33 ab | 0.94±0.28 ab | 0.87±0.06 a | 1.41±0.09 b |
| *Firmicutes* | 39.09±2.05 | 31.17±2.82 | 28.83±4.36 | 28.57±2.30 | 35.45±6.94 | 51.87±0.47 a | 51.97±4.95 ab | 52.39±12.63 ab | 39.93±0.97 b | 49.64±1.11 a |
| *Gemmatimonadetes* | 6.19±0.80 ab | 7.08±0.09 a | 5.60±0.44 ab | 5.59±0.35 b | 7.44±0.80 ab | 5.89±1.09 | 3.42±0.28 | 3.23±0.68 | 3.85±0.20 | 5.69±0.76 |
| *Ignavibacteriae* | 0.26±0.01 a | 0.32±0.03 ab | 0.35±0.01 b | 0.30±0.02 ab | 0.33±0.03 ab | 0.12±0.02 | 0.14±0.04 | 0.20±0.07 | 0.17±0.01 | 0.16±0.03 |
| *Nitrospirae* | 1.29±0.18 ab | 0.81±0.15 a | 1.34±0.14 ab | 1.34±0.14 ab | 1.47±0.01 b | 1.03±0.21 ab | 0.49±0.14 a | 0.90±0.28 ab | 0.92±0.14 ab | 1.20±0.06 b |
| *Planctomycetes* | 3.70±1.35 | 3.51±0.19 | 3.67±0.81 | 3.53±0.28 | 4.28±1.11 | 7.60±0.57 a | 1.50±0.07 c | 3.65±0.84 b | 4.06±0.22 b | 4.34±0.75 b |
| *Proteobacteria* | 23.79±1.05 | 25.05±0.54 | 25.59±0.80 | 24.83±0.97 | 22.58±2.35 | 16.62±0.41 a | 21.11±3.42 ab | 16.99±4.61 ab | 22.29±1.28 b | 19.00±0.38 b |
| *Unclass_Bacteria* | 1.27±0.11 | 1.12±0.09 | 1.16±0.06 | 1.18±0.06 | 1.26±0.10 | 0.87±0.05 a | 0.43±0.04 b | 0.54±0.16 ab | 0.68±0.08 ab | 0.92±0.03 a |
| *Verrucomicrobia* | 0.33±0.01 | 0.28±0.01 | 0.35±0.04 | 0.39±0.03 | 0.30±0.05 | 0.37±0.06 ab | 0.16±0.02 a | 0.25±0.09 ab | 0.28±0.01 b | 0.36±0.08 ab |

Data is presented as mean±SEM. Different letters indicate significant differences in relative abundances affected by soil treatments within site, *Tukey test*, *p < 0.05* and n = 3. Increased and decreased bacterial relative abundances in treated replant disease (RD) soils compared to untreated within site are highlighted in green and red, respectively. Colored cells indicate those changes that were found at both sites.

**Table S4: Fungal community diversity based on operational taxonomic units (OTUs) at 95% similarity in soils at two sites**

| **Site** | **Number OTU** | **Chao1** | **Shannon** |
| --- | --- | --- | --- |
| K | 104±3 a | 126±5 a | 2.78±0.08 a |
| A | 133±5 b | 151±7 b | 3.03±0.07 b |

Data is presented as mean±SEM. Letters indicate significant differences, *Tukey test*, *p* < *0.05* and n = 19. Chao1, species richness. Increased bacterial richness and diversity is highlighted in green.

**Table S5: Relative abundance of fungal phyla in replant disease soils at two sites affected by different treatments**

| **Phylum** | **Site K** | | | | | **Site A** | | | | |
| --- | --- | --- | --- | --- | --- | --- | --- | --- | --- | --- |
|  | **K_RD** | **K_Basamid** | **K_*B. juncea*** | **K_*R. sativus*** | **K_*Tagetes*** | **A_RD** | **A_Basamid** | **A_*B. juncea*** | **A_*R. sativus*** | **A_*Tagetes*** |
| *Ascomycota* | 69.55±0.69 | 66.01±6.45 | 66.02±0.94 | 62.86±3.16 | 64.82±3.65 | 43.38±6.14 | 68.14±6.76 | 51.19±3.50 | 60.74±2.15 | 53.95±0.49 |
| *Basidiomycota* | 4.38±0.55 ac | 2.06±021 b | 7.07±0.92 c | 16.18±1.65 d | 3.35±0.16 a | 15.71±1.19 a | 7.22±1.86 b | 19.52±4.08 ab | 19.06±2.95 ab | 19.63±2.35 a |
| *Chytridiomycota* | 0.15±0.05 | 0.13±0.04 | 0.08±0.02 | 0.11±0.05 | 0.20±0.05 | 0.46±0.16 | 0.31±0.11 | 0.35±0.10 | 0.22±0.06 | 0.41±0.07 |
| *Glomeromycota* | 0.00±0.00 a | 0.00±0.00 a | 0.00±0.00 a | 0.00±0.00 a | 0.09±0.04 b | 0.02±0.01 | 0.01±0.01 | 0.00±0.00 | 0.00±0.00 | 0.02±0.01 |
| *Rozellomycota* | 0.16±0.04 | 0.44±0.16 | 0.17±0.03 | 0.25±0.09 | 0.26±0.07 | 0.33±0.12 | 0.23±0.04 | 0.27±0.07 | 0.19±0.08 | 0.84±0.46 |
| *Unclass_Fungi* | 11.03±0.93 a | 18.39±4.41 ab | 8.74±0.34 ab | 6.53±0.80 b | 13.54±2.53 ab | 19.43±8.94 | 7.45±2.91 | 6.82±1.88 | 5.23±1.37 | 5.86±2.06 |
| *Zygomycota* | 14.72±1.94 | 12.97±4.64 | 17.92±0.90 | 14.08±2.96 | 17.74±1.86 | 20.66±3.90 | 16.64±9.88 | 21.85±2.27 | 14.55±1.84 | 19.29±1.63 |

Data is presented as mean±SEM. Different letters indicate significant differences in relative abundances affected by soil treatments within site, *Tukey test*, *p* < *0.05* and n = 4, except the soil treated with *Tagetes*, n = 3. Increased and decreased fungal relative abundances in treated replant disease (RD) soils compared to untreated within site are highlighted in green and red, respectively. Colored cells indicate those changes that were found at both sites.

**Table S6: Selected nematode population in analyzed soils taken in October 2013 (per 100 ml soil)**

| **Species** | **Site K** | | | | | **Site A** | | | | |
| --- | --- | --- | --- | --- | --- | --- | --- | --- | --- | --- |
|  | **K_RD** | **K_Basamid** | **K_*B. juncea*** | **K_*R. sativus*** | **K_*Tagetes*** | **A_RD** | **A_Basamid** | **A_*B. juncea*** | **A_*R. sativus*** | **A_*Tagetes*** |
| *Pratylenchus sp.* | 10.3±6.8 | n.d. | 16.0±17.4 | 19.7±13.9 | 4.0 | 77.0±53.4 | 11.3±5.8 | 90.7±83.3 | 89.3±86.4 | 11.0±2.8 |

Statistical test was not applied due to heterogeneity of nematode population within the treatment. Data is presented as mean±SD, n = 3. n.d., not detected. The number without ±SD, meaning the detection was found only in one replicate. RD, replant disease soil.

**List of supplementary figures**


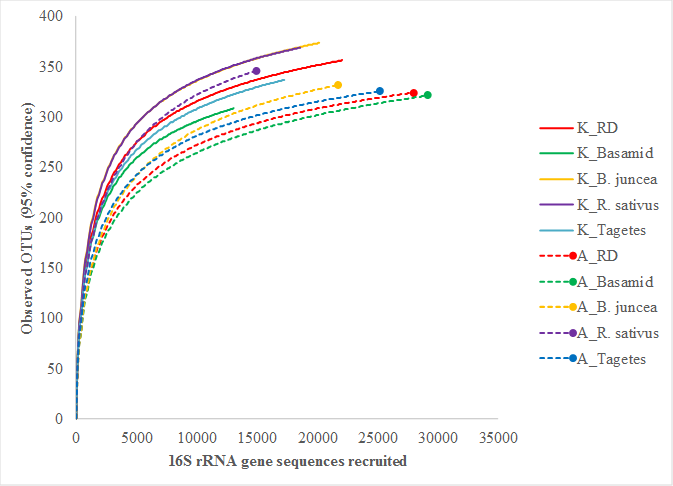


**Figure S1:** Rarefaction curves indicating the observed numbers of operational taxonomic units (OTUs) of bacterial communities in TC-DNA extracted from different soil treatments at sites K and A. RD, replant disease soil.


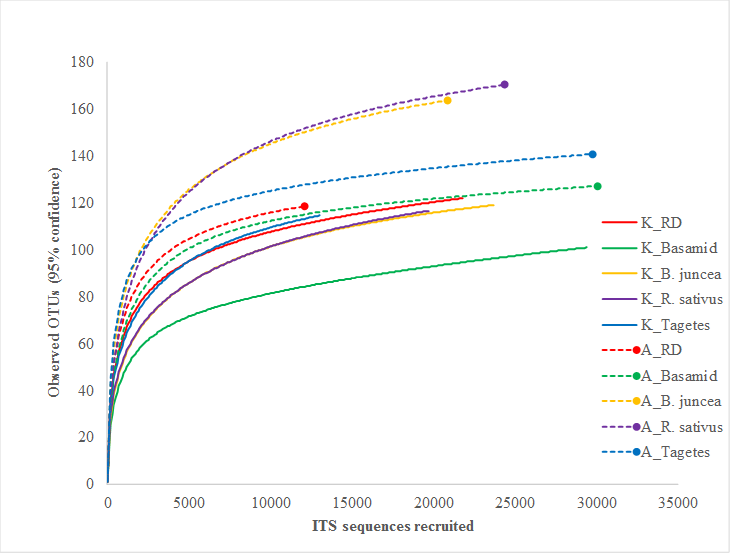


**Figure S2:** Rarefaction curves indicating the observed numbers of operational taxonomic units (OTUs) of fungal communities in TC-DNA extracted from different soil treatments at sites K and A. RD, replant disease soil.


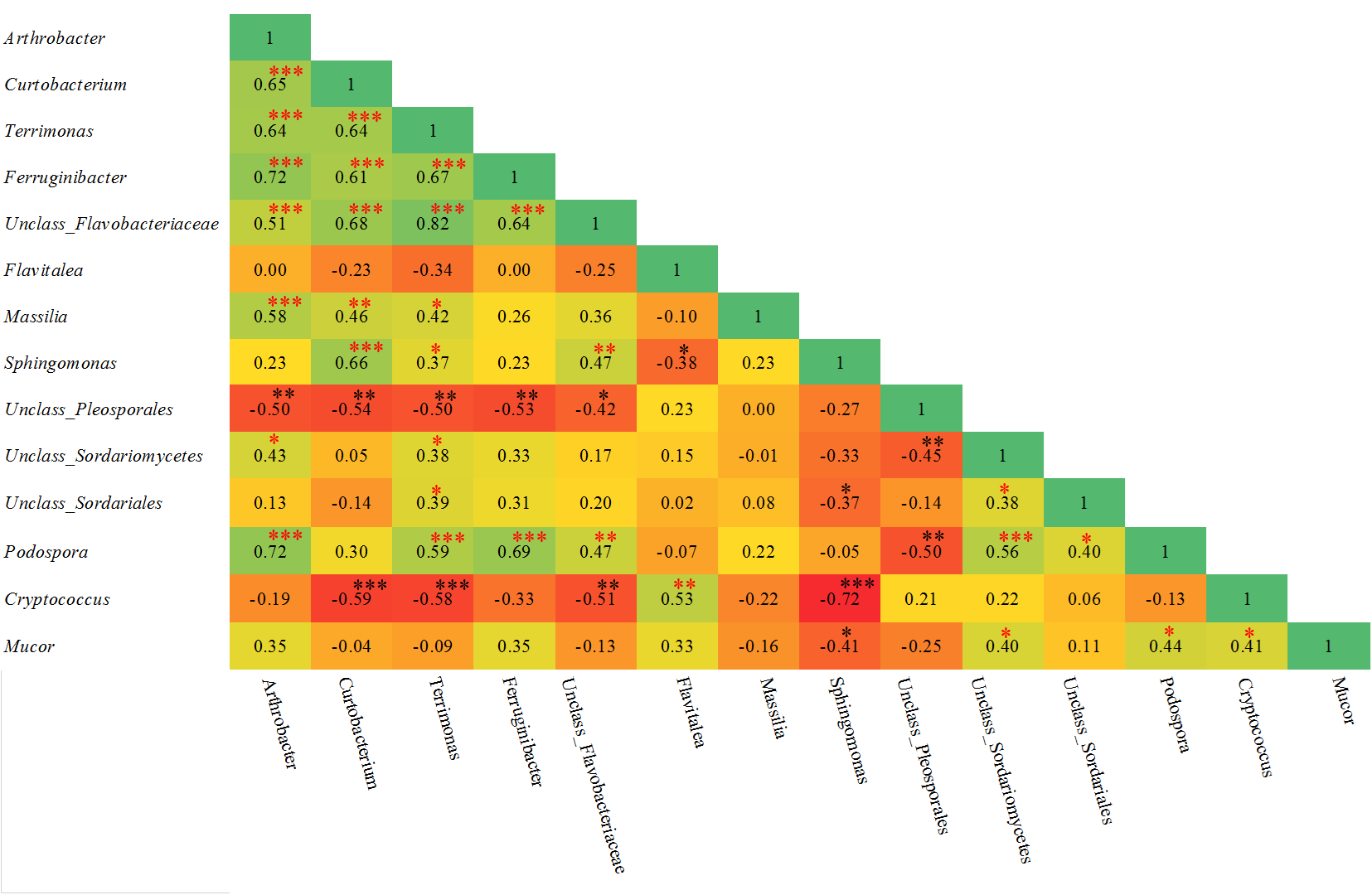


**Figure S3:** Spearman correlation coefficient (data in cells) between the relative abundance of the pairwise comparisons at the genus level, presented by the heat map. Asterisk indicates significant correlations between the pairs, *, *p* < 0.05; **, *p* < 0.01 and ***, *p* < 0.001. Past3 software.
